# A rare case of *Aerococcus urinae* infective endocarditis in an atypically young male: case report and review of the literature

**DOI:** 10.1186/s12879-018-3414-0

**Published:** 2018-10-17

**Authors:** Joseph M. Yabes, Serafim Perdikis, David B. Graham, Ana Markelz

**Affiliations:** 1 Brooke Army Medical Center, Department of Infectious Disease, 3551 Roger Brooke Drive, JBSA Fort Sam Houston, San Antonio, TX 78234 USA; 2 Brooke Army Medical Center, Department of Internal Medicine, 3551 Roger Brooke Drive, JBSA Fort Sam Houston, San Antonio, TX 78234 USA; 3 Brooke Army Medical Center, Department of Cardiothoracic Surgery, 3551 Roger Brooke Drive, JBSA Fort Sam Houston, San Antonio, TX 78234 USA

**Keywords:** *Aerococcus urinae*, Infective endocarditis, Aerococci

## Abstract

**Background:**

*Aerococcus urinae* is a gram-positive, alpha-hemolytic coccus bacterium primarily implicated in less than 1 % of all symptomatic urinary tract infections. Risk factors for disease include male gender, advanced age, and comorbid genitourinary tract pathology. Infections beyond the genitourinary tract are rare, though spondylodiscitis, perineal abscesses, lymphadenitis, bacteremia, meningitis, and endocarditis have been reported. Less than fifty cases of *A. urinae* infective endocarditis (IE) have been described in the literature. The rare occurrence of *A. urinae* in human infections and resultant lack of randomized controlled trials have resulted in a significant degree of clinical uncertainty in the management of *A. urinae* IE.

**Case presentation:**

We present an unusual case of a forty-three year-old male with *A. urinae* infective endocarditis (IE) who was successfully treated with mitral valve replacement and six weeks of penicillin/gentamicin therapy. In addition, we include a comprehensive review of all reported cases of IE due to *A. urinae* with specific attention to therapeutic regimens and treatment durations.

**Conclusion:**

Recent advances in diagnostic technology have led to an increase in the frequency *A. urinae* is diagnosed. Reviewing cases of *Aerococcus urinae* infections, their clinical courses and subsequent management can assist future healthcare providers and their patients.

## Background

The bacterial genus *Aerococcus* is comprised of gram positive, alpha hemolytic, catalase negative cocci. *Aerococcus* was first described as early as 1938, at that time initially thought to be a nonpathogenic contaminate and referred to as an “altered streptococci” [1]. It was not until in the early 1950’s that *Aerococcus* was comprehensively described and recognized as a single species [2]. In 1992 the *Aerococcus* genus was divided into *Aerococcus viridans* and *A. urinae* with the use of 16sRNA sequencing [3]. With improvements in microbiologic techniques and increased awareness of this entity there are now seven distinct *Aerococci* species. Two of these species, *Aerococcus urinaeequi* and *Aerococcus suis,* have not been found to occur in humans. *Aerococcus* species are ubiquitous in the environment: found in soil, air, and as part of the normal microbiota of certain mammals [4–6]. Despite this ubiquitous distribution, *A. urinae* is a rare cause of invasive infection. The incidence of *A. urinae* in human urinary tract infections is estimated to be 0.2–0.8% [4]. Invasive disease and bacteremia with *A. urinae* is likewise rare, cited to occur 0.5–3 cases per 1 million inhabitants per year [6, 7]. We present a case of *A. urinae* IE involving an atypically young patient. Furthermore, we include what we believe to be the most comprehensive review of previously reported episodes of *A. urinae* IE with the intent to aid clinicians with antimicrobial treatment regimens and duration.

## Case presentation

A forty-three year-old, active duty, Caucasian male presented to our hospital with a complaint of acute onset dyspnea. His past medical history included post-traumatic stress disorder, chronic migraines, and a recent admission for prostatitis approximately five weeks prior. He was an active duty officer in the US Army, who was a non-smoker, a non-drinker, and who denied illicit drug use. His previous admission had been complicated by urinary retention necessitating the placement of a foley catheter. Urine culture at that time resulted with ten thousand colony forming units of viridans group streptococci identified through colony morphology and biochemical testing. As part of the laboratory’s standard operating procedure susceptibility testing was not performed in the absence of a physician request due to a bacterial colony count less than one hundred thousand. He was subsequently discharged home with a fourteen day course of empiric Levofloxacin 500 mg once daily.

**Fig. 1:**
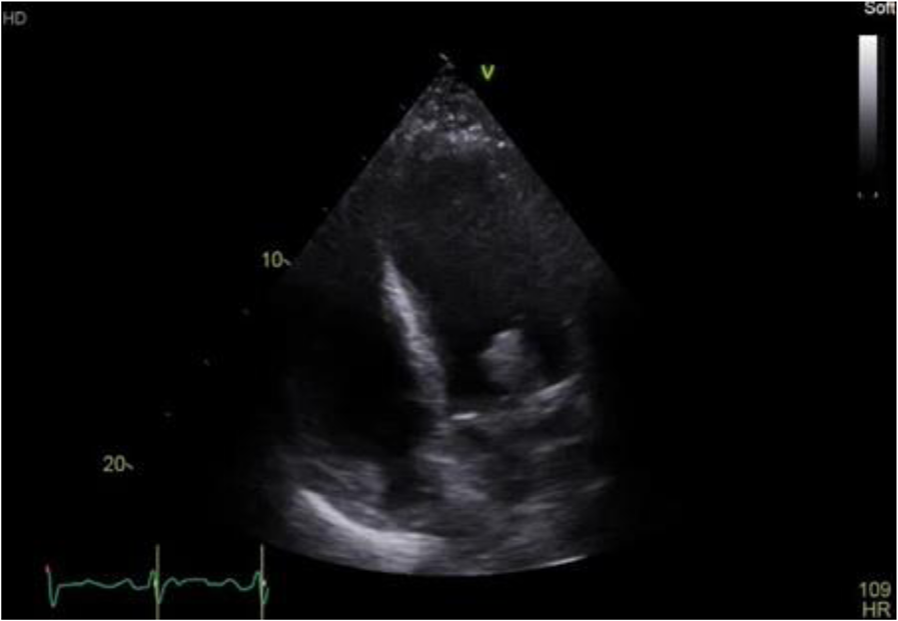
Transthoracic Echocardiogram revealing large, mobile vegetation on mitral valve

On re-presentation, he denied the presence of genitourinary symptoms. Initial vital signs revealed blood pressure of 120/73 mmHg, pulse rate of 140 beats per minute, temperature of 99.4 degrees Fahrenheit, respiratory rate of 34 breaths per minute, and oxygen saturation of 94% on room air. Physical examination was notable for mild respiratory distress with supraclavicular retractions, tachycardia with new 3/6 holosystolic murmur, and pitting lower extremity edema. There was no evidence of splinter hemorrhages, Janeway lesions, or Osler nodes. The remainder of the physical examination was within normal limits. Notable laboratory results were as follows: leukocyte count 13.3 × 10^3^, hemoglobin 8.1 g/dL, platelet count 150 × 10^3^, C - reactive protein 11.5 mg/dL, erythrocyte sedimentation rate 68 mm/hr., troponin of 0.08 ng/mL, and renal function panel with an anion gap of 18. Radiographic studies included portable chest x-ray and chest CT scan revealing pulmonary edema and bilateral pleural effusions. The patient was started on empiric vancomycin and piperacillin-tazobactam antibiotic therapy. Despite hemodynamic stability at presentation, his cardiopulmonary status deteriorated over the course of six hours until the patient required vasopressor support and eventual intubation. Bedside transthoracic echocardiogram revealed a large, pedunculated, highly mobile echo dense mass involving the anterior mitral leaflet measuring 2.2 cm × 1.7 cm with associated severe mitral regurgitation. (Fig. 1).

**Fig. 2:**
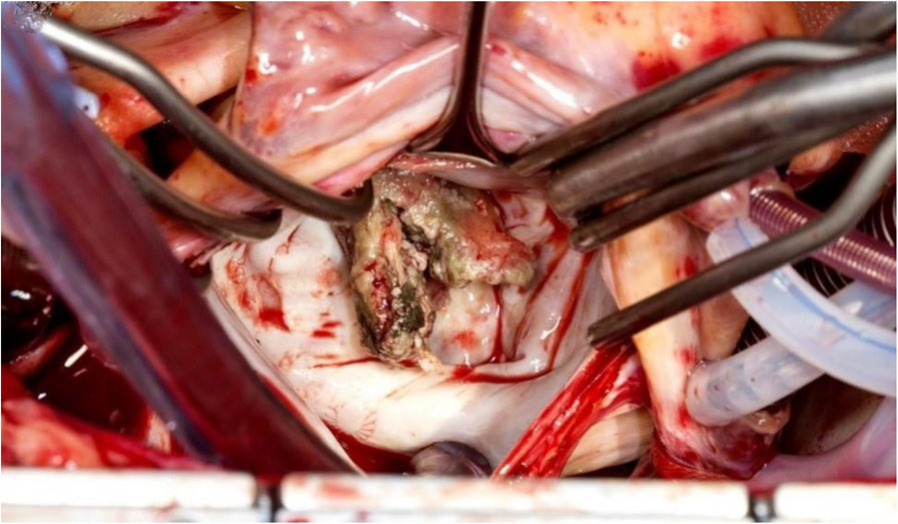
Intraoperative view of mitral valve vegetation on first inspection

**Fig. 3:**
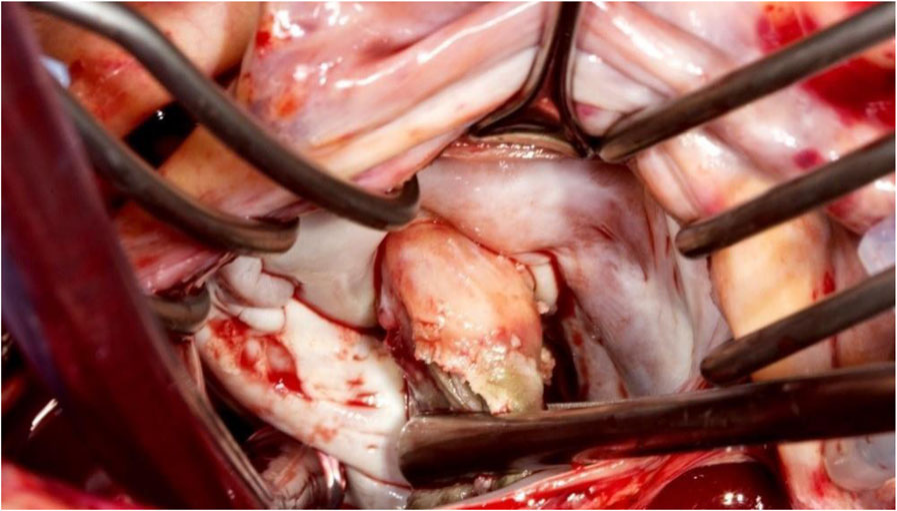
Vegetation manipulated forward, displaying firm attachment to the anterolateral commissure of mitral valve

**Fig. 4:**
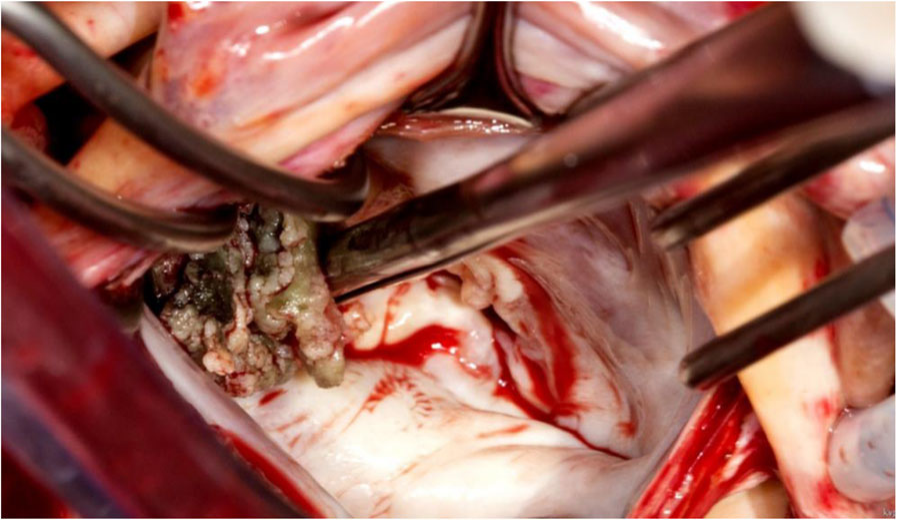
Mitral valve leaflets and chordae with architectural distortion from infection

Due to the patient’s hemodynamic instability from acute heart failure from newly discovered cardiac vegetation on the mitral valve, the cardiothoracic surgery service took the patient to the operating room for an emergent mitral valve replacement. Intraoperatively, it was discovered that the vegetation involved mainly the anterolateral commissure (A1/P1 leaflets) but also extended into P2 and P3. There was also infectious involvement beyond the valve into the chordae. (Figs. 2, 3 and 4) Extensive intraoperative debridement was undertaken, the chordae to the posterior third leaflet were preserved, and the mitral valve was replaced with a 31 mm St. Jude mechanical valve. Following his surgery, the patient was transferred to the cardiac intensive care unit for convalescence.

Blood cultures initially obtained in the Emergency Department grew gram-positive cocci in clusters at approximately thirty-six hours. Identification of the bacteria was later confirmed as *A. urinae* by use of the bioMerieux Matrix Assisted Laser Desorption Ionization Time Of Flight (MALDI-TOF) utilizing the Vitek MS database. Species identification was accepted after meeting the greater than 85 % confidence value threshold. Antibiotic susceptibilities obtained via agar diffusion revealed a penicillin susceptible strain with 0.12mcg/ml by ETEST (bioMerieux). Ceftriaxone and vancomycin susceptibilities were obtained via Kirby Bauer susceptibility testing and revealed intermediate 2mcg/ml and susceptible results 1mcg/ml, respectively. No additional antibiotic susceptibility testing was performed. Tissue culture performed on the cardiac vegetation itself resulted in identical identification and susceptibilities. Urine cultures failed to grow any pathogen. With susceptibility results known, the antibiotic regimen was narrowed to a continuous infusion of penicillin G dosed at twenty-million units over twenty-four hours combined with once daily gentamicin dosed at 3 mg/kg. The patient’s post-operative course was uneventful. He remained inpatient for an additional ten days while undergoing diuresis and awaiting his oral warfarin to reach a therapeutic level. His intravenous antibiotic regimen was continued for a total of six weeks from date of first negative blood cultures. Follow-up transthoracic echocardiogram obtained at the completion of antibiotic therapy displayed an appropriately functioning prosthetic valve and preserved ventricular systolic function. In addition to antibiotic therapy, the patient was treated with a six-week course of cardiac rehabilitation.

## Discussion and conclusions

On the rare occasions that *Aerococci* are encountered in human disease, they are predominantly implicated in urinary tract infections though invasive disease is known to occur. Overall incidence of IE due to *A. urinae* is unknown, but with increasingly sophisticated laboratory techniques the reported incidence of *A. urinae* is increasing [5, 8]. The clinical presentation of *A. urinae* IE is similar to the presentation of IE due to other bacterial etiologies. Fever, malaise, dyspnea (most often due to valvular dysfunction with ensuing pulmonary edema) and septic shock were common clinical manifestations of disease [5, 9]. The patient discussed above presented predominantly with signs and symptoms of acute pulmonary edema from valvular dysfunction. On extensive questioning, he denied subjective fevers, weight loss, and generalized malaise that might have led to an earlier diagnosis. This case of *A. urinae* IE involved a patient who was unusually young. Recognized risk factors for invasive *A. urinae* infection include male gender, age greater than sixty-five years, and pre-existing urinary tract pathology [5, 11–17]. On our review of the literature, there has been only one other reported case involving a patient who was younger at forty two years of age [18], and only one additional case involving a patient who was the same age [19].

In reviewing all cases published to date, we found the mean age of all patients affected to be 72 years and the mean age of male patients to be 73 years. Despite his atypically young age, he otherwise possessed the commonly associated urinary tract pathology. It is the author’s belief that the patient’s initial admission for prostatitis with traumatic foley placement led to the creation of a false urinary lumen with subsequent prolonged foley catheter placement and provided the opportunity for infection. Of all the forty-three cases found in the literature twenty-nine of them had documented urinary pathology. The remaining few either had a concurrent malignancy (4/43), hepatic disease (2/43), or pre-existing valvular disease (3/43). Unfortunately, there were an additional four patients where comorbid conditions were not discussed and only one patient where the authors specifically stated that there were no predisposing medical conditions to invasive disease [18]. Gritsch et al. state in their report of *A. urinae* IE that not only is *A. urinae* associated with urinary tract pathology but should instead be considered an opportunistic pathogen as their patient’s medical comorbidity was hepatic in nature [19].

Historically, *A. urinae* is considered to be an under recognized cause of human disease [4, 20, 21]. *A. urinae* is classically described on gram stain as being arranged in tetrads but also has been known to occur in clusters and irregular pairs. Gram stain identification alone, if not in classic tetrad morphology, may lead to misidentification as a staphylococcus species. Catalase negativity helps to distinguish aerococci from staphylococci. Catalase negativity may also cause the isolate to being mistaken for a streptococcal species. Alpha-hemolytic growth on blood agar may further contribute to this misidentification. The viridans group streptococci isolated from our patient’s initial urine culture may have similarly been misidentified. Mass spectrometry was not utilized and the low colony count may have led to an underappreciation of its significance. These phenotypic ambiguities on gross microscopic examination have likely contributed to the genus being under recognized and misidentified as has been noted previously [3, 20–22]. Biochemical methods have been employed in identifying *A. urinae*. Included in these are the API 20 STREP, ID 32 STREP, and Vitek 2 ID-GPC card (bioMerieux). In a study by Cattoir et al. in 2010 these commercial testing methods were able to correctly identify *A. urinae* in isolates obtained from urine cultures 100%, 95% and 45%, respectively [20]. In our facility once blood cultures become positive they are typically placed onto the MALDI-TOF, as well as the Vitek-2 for identification and susceptibilities, respectively. In the case discussed, the isolate itself was particularly difficult to culture on blood agar resulting in identification via MALDI-TOF and susceptibilities were obtained via Kirby Bauer method and Etest, rather than using the Vitek-2. The case presented exemplifies how MALDI- TOF has helped to overcome difficulties in identification. The diagnostic accuracy of MALDI-TOF and clinical usefulness in terms of identifying aerococcal infections have been previously well-cited [4, 5, 7, 16, 21].

The increasing rates of bacterial isolation provide treating physicians with a known etiology; however, they also present a clinical challenge to physicians. As identification of aerococcal infections increases, clinicians will find themselves faced with the question of what antimicrobials are most efficacious and what treatment duration is appropriate. Due to the current lack of controlled scientific trials and lack of formalized treatment guidelines, therapy is often empiric and guided by expert opinion. Due to the rarity that *A. urinae* is clinically encountered, the Clinical and Laboratory Standards Institute (CLSI) has only recently be able to add microbiology workup and breakpoints to their guidelines [23]. In an effort to review the treatment strategies other clinicians and the associated outcomes, we have compiled all available reported cases of *A. urinae* IE (Table 1). To our knowledge there have been less than fifty total cases of *A. urinae* IE reported and this represents the most comprehensive review to date. We did find an additional three reports not included in Table 1, but the manuscripts were not available in English and therefore not included. Cases of IE due to Aerococcus-like organisms (ALOs) were likewise excluded. It is important to note that previous reports of bacteremia, septicemia, and infective endocarditis exist due to ALOs. Furthermore, these cases are likely, at least a portion, attributable to *A. urinae* but categorized as ALOs either due to the limitations of diagnostic testing at that time, lack of recognition of *A. urinae* as a unique bacterial species at the time or both [24].

**Table 1:** Reported cases of Aerococcus urinae endocarditis

Case No.	Age (yrs)	Sex	Risk Factors	Valve	Surgery (y/n)	Antibiotic Regimen	Duration	Survived(y/n)	Ref No.
1	69	M	Cystoscopy	Av	N	β Lactam/AG	6wks	Y	[13]
2	54	M	Phimosis	Mv	Y	β Lactam	6wks	Y	[32]
3	43	M	Hepatitis C	Av	N	β Lactam/AG	5 days	N	[19]
4	68	M	Indwelling Catheter	Av	Y	β Lactam/AG	6/2wks	Y	[29]
5	80	M	Malignancy	Av	Y	β Lactam	6wks	Y	[33]
6	77	M	BPH	Av	N	β Lactam/Van	–	N	[10]
7	68	M	BPH	Mv	N	β Lactam/AG; oral regimen	3wks; unknown	Y	[14]
8	75	M	Cystoscopy	Av	Y	β Lactam/AG	6wks	Y	[15]
9	89	M	TURP	Mv	N	β Lactam/AG	7 days	N	[16]
10	81	M	UTI	Av	N	β Lactam/AG	2wks/8da	N	[34]
11	42	M	None	Av	Y	β Lactam/AG	6wks	Y	[18]
12	49	M	–	Av	–	β Lactam/AG	–	Y	[35]
13	54	M	Urethral Stricture	T/Av	N	β Lactam/AG	–	N	[36]
14	69	M	Malignancy	Av	Y	β Lactam	12wks	Y	[17]
15	62	M	BPH	M/Av	Y	β Lactam/Rif, AG	6wks, AG 2wks	Y	[25]
16	78	M	Aortic stenosis	Av	N	β Lactam/AG	10 days	N	[26]
17	74	M	BPH	Mv	Y	β Lactam/AG	4wks	N	[11]
18	81	M	BPH	Mv	N	β Lactam/AG	6wks	Y	[12]
19	78	M	Indwelling Catheter	Av	N	β Lactam	–	N	[12]
20	87	M	BPH	Mv	N	–	–	N	[12]
21	78	F	Ureteral Stent	Av	N	β Lactam/Van	2wks	N	[12]
22	48	M	ASD	Mv	N	β Lactam/AG	–	Y	[27]
23	79	F	UTI	Av	N	β Lactam	6wks	Y	[27]
24	91	M	Indwelling Catheter	Mv	–	β Lactam/AG	4wks/10d^b^	Y	[5]
25	91	M	BPH	Mv	–	β Lactam/AG	4wks/10d^b^	Y	[5]
26	89	F	–	Mv	–	β Lactam/AG	4wks/10d^b^	Y	[5]
27	86	M	Urethral Stricture	Av	–	β Lactam/AG	4wks/10d^b^	Y	[5]
28	83	M	Urethral Stricture	Mv	–	β Lactam/AG	4wks/10d^b^	Y	[5]
29	80	F	–	Mv	–	β Lactam/AG	4wks/10d^b^	Y	[5]
30	77	M	–	Av	–	β Lactam/AG	4wks/10d^b^	Y	[5]
31	75	M	BPH	Mv	–	β Lactam/AG	4wks/10d^b^	Y	[5]
32	74	M	Suprapubic Catheter	–	–	β Lactam/AG	4wks/10d^b^	Y	[5]
33	65	M	Indwelling Catheter	Mv	–	β Lactam/AG	4wks/10d^b^	Y	[5]
34	53	M	Dysuria	Av	–	β Lactam/AG	4wks/10d^b^	Y	[5]
35	49	F	Intermittent Catheter	Av	–	β Lactam/AG	4wks/10d^b^	Y	[5]
36	81	M	Indwelling Catheter	Mv	–	β Lactam/AG	4wks/10d^b^	Y	[5]
37	74	F	Malignancy	–	–	β Lactam/AG	4wks/10d^b^	Y	[5]
38	87	M	Malignancy	Mv	–	β Lactam/AG	**–**	N	[9]
39	77	M	Liver Failure	Av	–	β Lactam/AG	**–**	Y	[9]
40	83	M	Indwelling Catheter	Mv	–	β Lactam/AG	**–**	Y	[9]
41	73	M	Suprapubic Catheter	–	–	β Lactam/AG	**–**	N	[9]
42	88	F	Aortic Stenosis	Av	–	β Lactam/AG	**–**	Y	[9]
43	43	M	Indwelling Catheter	Mv	Y	β Lactam/AG	6wks	Y	^a^

*β Lactam/AG* Beta-Lactam/Aminoglycoside, Van-vancomycin, *BPH* benign prostatic hyperplasia; *ASD* Atrial septal defect, *TURP* trans-urethral prostate biopsy, *UTI* urinary tract infection, ^a^This paper, ^b^Median Duration of therapy for each agent

Treatment regimens for *A. urinae* IE have largely relied on beta-lactams with or without synergistic aminoglycoside usage. However, this appears in large part to be done empirically with broad-spectrum regimens narrowed after local laboratory susceptibility testing was completed [5, 9, 10, 12–14, 16, 19, 25–27]. In vitro studies regarding the antibiotic susceptibilities of *A. urinae* isolates have shown susceptible MIC’s to most beta-lactams employed in IE. Fluoroquinolone resistance has likewise been previously reported [28]. The clinical relevance of this is magnified when taking into account the common usage of fluoroquinolone therapy aimed at treating urinary tract infections which is presumably the initial nidus of infection. The patient presented was similarly treated with empiric Levofloxacin therapy. While there remains some question as to whether the original isolate was misidentified this is speculation only and we unfortunately have no way to verify speciation or susceptibility testing. *A. urinae* is also inherently resistant to sulfonamides and previously thought to have similar inherent resistance to trimethoprim; though recently, the methodology regarding the media used – where trimethoprim resistance has been observed – has been implicated with changing the result [23, 28]. Durations of synergistic aminoglycoside varied from ten days to six weeks. Synergistic effect on *A. urinae* isolates have been observed via in vitro studies. Though this is not universal, in one study by Sunnerhagen et al. approximately half of the *A. urinae* isolates tested failed to display a synergistic effect of combination beta-lactam gentamicin therapy [5].

The largest case series of *A. urinae* IE treated fourteen patients with a median duration of ten days of aminoglycoside therapy and four weeks of beta-lactam therapy [5]. Specifics on duration of follow up or re-hospitalization rates were not addressed, but this series suggests that a shorter duration of therapy may be efficacious with the right patient population. Of patients who experienced favorable response to therapy, the shortest duration of therapy employed was three weeks. We chose six weeks of combination therapy due to the placement of a mechanical mitral valve and after reviewing treatment strategies for similar cases of IE. The patient voiced his desire for both the longer duration as well as combination therapy, which also influenced our final treatment regimen. Death rates of *A. urinae* IE were previously thought to be increased compared to IE due to other infective etiologies [14, 16, 29]. Overall mortality of *A. urinae* IE has since been shown to be equivalent to that of other etiologies and previous reports were likely skewed due to the tendency of case reports to focus on the dramatic and spectacular [5].

Our review of reported cases of *A. urinae* endocarditis showed that 12/43 cases (27%) resulted in death (Table 1). Of note, only one of those twelve patients had an operation. Given the older ages and multiple comorbidities of the patients typically afflicted by *A. urinae* IE, it is possible that many did not receive surgical therapy due to their unfavorable risk profile or they did not meet Class I surgical indications as outlined by the 2014 AHA/ACC Guideline for the Management of Patients with Valvular Heart Disease [30]. Surgical intervention was successfully performed in 9/43 cases (21%) of which only one patient did not survive.

We have presented a rare case of *Aerococcus urinae* infective endocarditis in an uncharacteristically young patient. To our knowledge, we have also compiled the most extensive case review of *Aerococcus urinae* endocarditis. In the absence of controlled clinical trials it is the author’s opinion that, if it can be safely accomplished, patients should be treated with six weeks of antimicrobial therapy with combination aminoglycoside antibiotics. In the case presented, we opted to treat utilizing a continuous infusion of penicillin. We supposed that if *A. urinae* is commonly mistaken for viridans group streptococci, then perhaps previous cases of viridans group streptococci IE were actually due to *A. urinae*. Current guidelines for the treatment of viridans group streptococci IE support continuous infusion penicillin therapy [31]. Continuous infusion of a beta-lactam antibiotic has been reported as effective in at least one previous report of *A. urinae* IE and allowed us to maximize the time above minimal inhibitory concentration [12]. Furthermore, it is our opinion that for outpatient administration it is perhaps more practical and convenient. Future multi-centered studies are needed to investigate both the optimal duration of therapy and patient outcomes with and without synergistic aminoglycoside antibiotic therapy. In the absence of such studies, it is our hope that this review of will assist future clinicians in the care of their patients.

## Abbreviations

ALOs: Aerococcus-like organisms
CLSI: Clinical and Laboratory Standards Institute
IE: Infective endocarditis
MALDI-TOF: Matrix Assisted Laser Desorption Ionization Time Of Flight
